# Alternative instrument for the evaluation of handgrip strength in Duchenne muscular dystrophy

**DOI:** 10.1186/s12887-022-03388-x

**Published:** 2022-06-10

**Authors:** Mariana Angélica de Souza, Edson Zangiacomi Martinez, Elisângela Aparecida da Silva Lizzi, Ananda Cezarani, Gabriela Barroso de Queiroz Davoli, Marjory Irineu Bená, Cláudia Ferreira da Rosa Sobreira, Ana Claudia Mattiello-Sverzut

**Affiliations:** 1 Department of Health Science, Ribeirao Preto Medical School, University of São Paulo, Av. Bandeirantes, 3900, Campus, Ribeirão Preto, SP 14049-900 Brazil; 2 Department of Social Medicine, Ribeirao Preto Medical School, University of São Paulo, Ribeirão Preto, SP Brazil; 3 Department of Mathematics, Federal Technological University of Paraná, Cornélio Procópio, Paraná Brazil; 4 Department of Neurosciences of the Ribeirao Preto Medical School, University of São Paulo, Ribeirão Preto, SP Brazil

**Keywords:** Dynamometer, Handgrip strength, Duchenne muscular dystrophy

## Abstract

**Background:**

The commonly used dynamometers can be ineffective in evaluating handgrip in patients with Duchenne muscular dystrophy (DMD), especially children with generalized muscle weakness. The aim of this study was to analyze whether the modified sphygmomanometer is an effective instrument for handgrip strength evaluation in patients with DMD, during different stages of the disease.

**Method:**

The handgrip strength of 33 patients was evaluated by the Jamar dynamometer and the modified sphygmomanometer. Motor function was evaluated by the Motor Function Measurement (MFM) scale. Four evaluations, with a six-month interval between each, were performed: Evaluation 1 (*N* = 33), Evaluation 2 (*N* = 24), Evaluation 3 (*N* = 15), and Evaluation 4 (*N* = 8). A linear regression model with mixed effects was used for the longitudinal data and descriptive analysis of strength for all four evaluations.

**Result:**

The first evaluation data presented very high correlations between the dynamometer and the modified sphygmomanometer (*r* = 0.977; *p* < 0.001). The longitudinal analysis showed a significant difference between Evaluation 1 and the other handgrip strength evaluations obtained using the dynamometer (*p* < 0.05) but not the modified sphygmomanometer (*p* > 0.05). Null values were obtained only when using the dynamometer device.

**Conclusion:**

The modified sphygmomanometer seems to be more suitable than the dynamometer for measuring handgrip strength in all stages of DMD.

## Introduction

The natural progression of Duchenne muscular dystrophy (DMD) leads to increasing weakness and musculoskeletal deformities, occurring in a proximal to distal direction with the pelvic girdle being affected earlier than the shoulder girdle [1, 2]. Ambulation loss can happen from the age of nine [3], when the upper limbs become essential to activities such as wheelchair transfers and locomotion. There is no cure for this myopathy. All treatments are palliative and aim to control symptoms, enable the patient / family unit to adapt to the progress of the disease, and keep the patient as active as possible [4]. Thus, the dexterity of hands, which is of utmost importance for handling entertainment device tools such as those used for video games and computers, can improve the quality of life of people with disabilities such as DMD [5].

Nevertheless, the progressive weakness and loss of strength also affect the dexterity needed for reaching, grasping, and prehension. Abnormal dexterity, or incoordination, is observed in people with injuries involving the central nervous system [6] and myopathies [7], leading to pathological synergies and atypical postures [6]. According to the literature, different types of dynamometers have been used to assess grip strength, such as hydraulic (the Jamar dynamometer) [8], pneumatic (the bulb dynamometer, Martin Vigorimeter, modified sphygmomanometer) [9–12], and electronic (the Myogrip) [13, 14]. The Jamar dynamometer (here called “dynamometer”) is considered the gold standard of handgrip strength evaluation [8, 11]. This instrument evaluates the grip force through the digital hook-up, during which the interosseous muscles are the most recruited, while the thumb has minimal participation in this action [15]. Mattar et al. [16] analyzed the grasping response with the dynamometer in young DMD patients and observed a lower grip strength development in this population when compared to typical children. The authors also found a positive correlation between grip strength and higher degree of physical disability in the limbs, measured by the Brooke scale. However, the dynamometer has low validity in weakness detection for patients in different stages of myopathic disorders [17], as it is considered heavy, weighing about 2.5 kg [18], and ineffective at measuring strength in children with small variations in handgrip [19]. Another negative aspect of this portable device is its high price (around U$560.00), which makes it unaffordable for clinical practitioners.

As an alternative, the bulb dynamometer evaluates grip strength using the opposition between thumb and fingers. It is small in size and lightweight, which makes it ideal for evaluating typical children [9]. Our research group evaluated the grasping response, using the bulb dynamometer, in ambulatory patients with DMD and observed a very stable grip strength response, regardless of age [9]. This finding was different from what was observed in typical children, whose grip strength increased with increase in age, whereas a few instances of zero grip strength were observed in children with DMD using the bulb dynamometer. Children with DMD, at six and 10 years of age, achieved 79 and 50% of normal grip strength, respectively [9, 10].

Patients with hand weakness may present difficulties and/or inability to use the dynamometer. In this case, a pneumatic equipment is indicated to evaluate their muscle strength [11]. The modified sphygmomanometer is a portable pneumatic device, adapted from an aneroid sphygmomanometer,that engages all hand structures [20]. This instrument has been used in adults with Parkinson’s disease, and it has demonstrated good reliability and validity, as a means to compare them to typical adults [21, 22]. In contrast, the dynamometers most commonly used in the literature do not seem to meet the requirements for grasping that are observed in DMD patients, especially those with reduced mobility.

We hypothesize that the modified sphygmomanometer is an effective alternative for the evaluation of handgrip strength in children who are in different stages of the disease and present generalized muscle weakness. This study aims to verify whether the modified sphygmomanometer is a suitable device for measuring handgrip strength in ambulatory and non-ambulatory DMD patients, comparing it with the dynamometer, using a longitudinal and cross-sectional approach. The secondary aim is to explore the influences of age and motor function in the longitudinal responses obtained with these devices.

## Methods

### Ethical approval and consent to participate

The research work was performed in accordance with the Declaration of Helsinki and the National Council of Health (CNS), resolution 466/2012. The parents, and/or guardians of the patients, agreed to their participation by signing an informed consent form. This study was approved by the Ethics Committee of the Clinics Hospital of the Ribeirão Preto Medical School, University of São Paulo (HCFMRP-USP -process number 15508/2016).

### Sample

This study investigated a convenience sample of 33 patients from the Rehabilitation Center in the Clinics Hospital of the Ribeirão Preto Medical School, University of São Paulo (HCFMRP-USP). Both ambulatory patients (*N* = 21) and non-ambulatory patients (*N* = 12) were between six and 13 years of age at the first evaluation. To participate in the study, the patients needed to a) be diagnosed with DMD, b) be able to understand simple instructions, and c) have some measurable degree of handgrip strength (i.e., a handgrip strength different from zero) in at least one item of equipment used in this study. The DMD diagnosis was given based on the absence of dystrophin, as seen in the muscle biopsy and/or mutation identification of the dystrophin gene. Exclusion criteria were a) absence of a confirmed DMD diagnosis, b) history of fractures or upper limb surgeries, c) inability to understand simple instructions, and d) presence of muscle shortening that would prevent the positioning needed for prehension.

### Evaluation procedures

Patients were evaluated longitudinally and the following data were registered: body weight (kg), height (m), hand dominance, handgrip strength, and function. Four evaluations were performed with a six-month interval between each: Evaluation 1 (Ev1, *N* = 33), Evaluation 2 (Ev2, *N* = 24), Evaluation 3 (Ev3, *N* = 15), and Evaluation 4 (Ev4, *N* = 8). Our N decreased over time due to patients missing the evaluations. The evaluations were performed by two different trained researchers.

The body weight was obtained using an electronic anthropometric scale (Welmy® W200 / 5). The values were recorded in kg and fractions of 0.1 kg, according to the resolution of the scale. Wheelchair users were weighed with and without the wheelchair; when the wheelchair was used, its weight was subtracted from the final weight. The height was obtained by means of a tape measure and recorded in meters (m). For wheelchair users, the height was obtained by measuring the length of the arms or wingspan, using a horizontal stadiometer and metal tape, and the data were recorded in centimeters (cm), with an accuracy of 0.1 cm [23, 24]. The Body Mass Index (BMI) was calculated by the weight ratio, using height squared. Dominance was determined by asking the following question to the parent, caregiver, or patient: “Which hand do you write with?” The Motor Function Measurement scale (MFM), was used, according to the manual instructions, to evaluate motor function. It is composed of three dimensions and a total of 32 items, scored from 0 to 3 according to the patient’s performance in the execution of the tasks: Dimension 1 (D1): standing position and transfers (13 items); Dimension 2 (D2): axial and proximal motor function (12 items); and Dimension 3 (D3): distal motor function (7 items) [25].

**Fig. 1:**
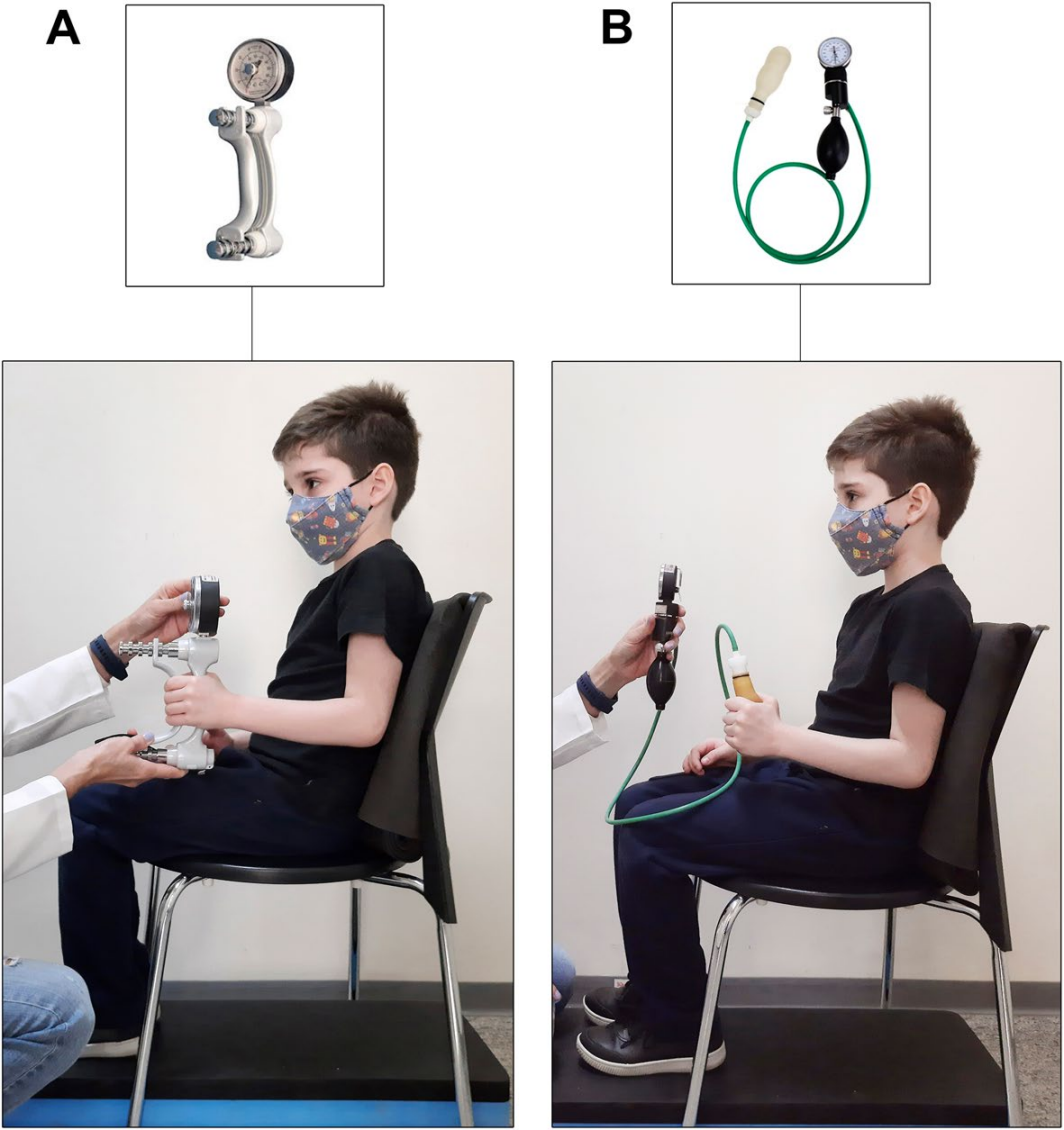
Standardized position for evaluation handgrip strength with dynamometer (**A**) and modified-sphygmomanometer (**B**)

The handgrip strength was evaluated by the dynamometer and the modified sphygmomanometer. Three handgrip strength measurements of the dominant hand were collected on both devices, with an interval of approximately 10 s between the three repetitions and approximately 3 min between the devices (starting with the dynamometer). The strength measurements were collected in a standardized position, according to the American Society of Hand Therapists (ASHT) [23]: patient sitting with back against the chair, feet flat on the floor, upper limb with shoulder adducted, neutral rotation, elbow in 90° flexion, neutral forearm, and wrist between 0° and 30° extension and between 0° and 15° ulnar deviation (Fig. 1A and B). The mean of the three measures was used for statistical analysis. Handgrip strength values, obtained with the modified sphygmomanometer in units of millimeters of mercury (mmHg), were converted to pound per square inch (psi), considering that 1 psi equals 51.75 mmHg. Handgrip strength values obtained with the dynamometer were recorded in units of kilogram force (Kgf).

### Statistical analysis

Initially, a descriptive analysis of the data was performed using the means and standard deviations. Correlation between dynamometer and sphygmomanometer measures at the first evaluation (*N* = 33) was calculated following adjustment for repeated observations, as described by Bland and Altman (1995) [26], where correlations of ≤0.19 = very low, 0.20–0.39 = low, 0.40–0.69 = moderate, 0.70–0.89 = high, and 0.90–1.0 = very high [27].

Regression models were used for the longitudinal analysis of evaluations 1, 2, 3, and 4. The handgrip strength data obtained with the dynamometer and modified sphygmomanometer during evaluations 1 to 4 were analyzed using the linear regression model with mixed effects. From each device over time, the estimated means were also compared to find evidence of difference. For this analysis, the age and MFM dimension 3 (D3) were covariables in the model. A residual analysis was conducted to check the adequacy of the model (assumptions of linearity, normality of residuals, and homoscedasticity of error variances, as well as outliers and influential cases). The level of significance was set at 5% for all analyses, and SAS (version 9.2) software, specifically the PROC MIXED, was used.

## Results

### Cross-sectional analysis

Age and anthropometric and functional variables are shown in Table 1. There was no data exclusion, as all patients met the inclusion criteria. The mean handgrip strength was 5.2 kgf (SD = 0.72) when evaluated by the dynamometer and 3.4 psi. (SD = 0.16), when evaluated by the modified sphygmomanometer (Table 1). Null values were only observed when the dynamometer device was used (19 null values from 80 evaluations). A very high correlation was observed between the dynamometer and the modified sphygmomanometer at the first evaluation (*r* = 0.977; *p* < 0.001).

**Table 1:** Characterization of the study sample and data acquisition for all four evaluations

Variables analyzed	Evaluation 1 (***n*** = 33)	Evaluation 2 (***n*** = 24)	Evaluation 3 (***n*** = 15)	Evaluation 4 (***n*** = 8)
**Age (Years)**	10.0 (0.33)	10.8 (0.34)	11.3 (0.35)	12.2 (0.37)
**Weight (Kg)**	37.2 (2.4)^a,b,c^	41.9 (2.51)	45.6 (2.77)	48.9 (4.7)
**Height (m)**	1.35 (0.03)	1.41 (0.03)	1.42 (0.04)	1.42 (0.07)
**BMI (Kg/m**^**2**^**)**	19.7 (1.2)	20.8 (1.2)	22.8 (1.6)	23.8 (3.6)
**MFM D1 (%)**	41.5 (6.2)^b,c^	34.8 (6.4)	26.8 (6.8)	25.0 (7.7)
**MFM D2 (%)**	87.9 (2.9)^c^	86.7 (3.0)^e^	86.0 (3.2)^f^	81.1 (3.4)
**MFM D3 (%)**	87.4 (2.0)	88.4 (2.2)^e^	87.6 (2.4)	82.4 (2.9)
**MFM Total (%)**	67.3 (3.6)^b,c^	65.7 (3.7)^e^	62.3 (3.9)	58.7 (4.2)
**Dynamometer (kgf)** **[95%CI]**	5.2 (0.72)^a,b,c^ [3.7 – 6.7]	3.8 (0.73)^d^ [2.4 – 5.3]	3.1 (0.8) [1.6 – 4.6]	3.0 (0.9) [1.2 – 4.8]
**Modified-sphygmo (psi)** **[95%CI]**	3.4 (0.16) [3.0 – 3.7]	3.3 (0.16) [3.0 – 3.6]	3.4 (0.17) [3.0 – 3.7]	3.6 (0.19) [3.2 – 4.0]

Mean values and standard errors (between brackets)

*n* number of patients, *BMI* body mass index, *MFM* measure of motor function, *D1* dimension 1 of MFM, *D2* dimension 2 of MFM, *D3* dimension 3 of MFM, 95%CI (95% confidence interval). Differences of least squares means (mixed effect models), *p* < 0.05 = a: Evaluation (Ev) 1 vs Ev2, b: Ev1 vs Ev3, c: Ev1 vs Ev4, d: Ev2 vs Ev3, e: Ev2 vs Ev4, and f: Ev3 vs Ev4

### Longitudinal analysis

**Fig. 2:**
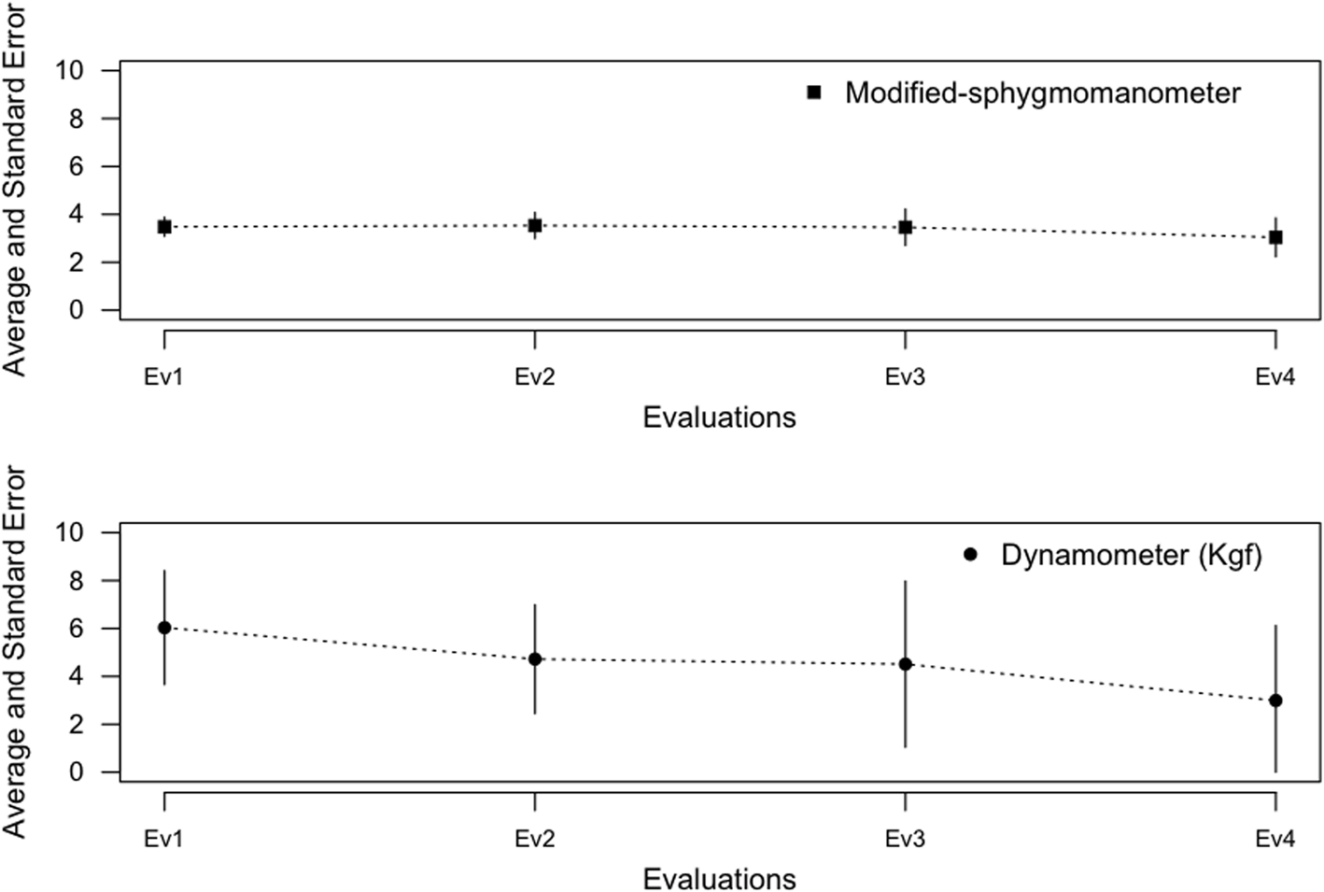
Evolution of mean values and 95% confidence interval for handgrip strength using the Jamar dynamometer and Modified-sphygmomanometer

The comparison of the evaluations carried out over time is shown in Table 1. An increased body weight and decreased score to MFM can be observed at D1, D2, D3, and Total over time. Grip strength, using the dynamometer, was significantly different between the evaluations (Ev1 vs Ev2, Ev1 vs Ev3, Ev1 vs Ev4, Ev2 vs Ev3; *p* < 0.05). The average of evaluation 1 was greater than that obtained in evaluations 2, 3, and 4. No evidence of statistical differences was observed for hand grip strength when the modified sphygmomanometer was used (*p* = 0.33) (Table 1). Additionally, the absolute difference between Ev1 vs Ev4 was 2.22 kgf for the dynamometer (95%IC 0.92 to 3.52, *p* < 0.01, t-value 3.38) and − 0.25 psi for the modified sphygmomanometer (95%IC − 0.53 to 0.03, *p* 0.08, t-value − 1.76). For the dynamometer, the absolute difference between Ev1 vs Ev2 was 1.40 kgf (95%IC 0.73 to 2.06, *p* < 0.01, t-value 4.16), and between Ev1 vs Ev3 it was 2.10 kgf (95%IC 1.24 to 2.96, *p* < 0.01, t-value 4.83). The evolution of handgrip strength during the four assessments, using both devices, is illustrated in Fig. 2. The estimated difference was lower for the modified sphygmomanometer in all three evaluations than the results obtained with the dynamometer (Table 1; Fig. 2).

## Discussion

A progressive reduction of upper limb muscle strength may compromise motor performance in daily functional activities of patients with DMD [15]; therefore, adequate grip measurement should be used for more effective therapeutic interventions. The results of this study indicate a very high correlation between the grip strength assessed by the modified sphygmomanometer and dynamometer, corroborating the findings of Vermeulen et al. (2015), who reported a strong correlation between hydraulic and pneumatic instruments [28]. Thus, it can be suggested that, if hydraulic and pneumatic equipment provides values that have a clinical correlation, professionals should opt for the device that allows the most reliable measurement and has the lowest price.

Our results show a significant difference in strength among the four evaluations made using the dynamometer but not those made using the modified sphygmomanometer. Null values were observed when the dynamometer device was used, independent of age. For older children and those in a more advanced phase of the disease, the dynamometer may indicate low results, due to the rigidity and weight of the instrument [11]. This seems to be the case in the present study. For patients aged 10, which was the average age of the sample, the grip strength assessed with the dynamometer confirms the lower grip strength development reported by Mattar et al. [16]. Compared to typical children, patients with DMD reach the highest percentage (normative value) for age 10 using the modified sphygmomanometer (50.7%) rather than the dynamometer (40.8%) [8, 9]. Thus, we can infer that the sphygmomanometer grip allowed for a complete grip, using all fingers, unlike the dynamometer, suggesting that grip strength might be underestimated in the latter.

The longitudinal data seem to confirm our hypothesis that the modified sphygmomanometer is effective for evaluation of handgrip strength in different stages of DMD. However, the data obtained with the dynamometer indicate a reduction in handgrip strength in all evaluations (every 6 months). Based on what is available in the literature, we did not expect this change for the following reasons: (1) Reduction in handgrip strength is not expected in younger patients [16]; (2) The change in grip strength (absolute values) during the period of 1 year is only noticeable for non-walking patients, with a mean age of 13.9 years [29]; (3) There is a strong correlation between muscle strength and motor function, evaluated by the MFM total score [13, 30]; and (4) In the initial stage of the disease, the upper limb functional capacity is preserved [10, 13, 31]. Thus, the patients are not expected to have reduced grip strength.

In the present study, using the modified sphygmomanometer, we observed the evolution of handgrip strength expected for the age of the patients and the two-year duration of the study. Taking into consideration the age of our patients (mean of 10 years) and the fact that the grip strength, assessed by the modified sphygmomanometer, did not change over 18 months (absolute values), we suggest that the modified sphygmomanometer provides more accurate data than the dynamometer. Moreover, we observed a significant fluctuation of grip strength when the dynamometer was used. This finding indicates a certain disparity of results between instruments, especially when we take motor function data into consideration. From the biomechanical point of view, the dynamometer stimulates a hook-and-loop pattern, characterized by thumb and digits in adduction, flexed fingers, a flat arch, and a narrow range of digits, which, according to Kapandji (2000) [15], does not favor the grip because it does not fully adapt to the shape of the object. In contrast, the cylindrical grip exerted on the modified sphygmomanometer is characterized by volar flexion, a neutral position of the wrist, thumb in abduction and opposition, and fingers in abduction and flexion. Still, according to Kapandji (2000) [15], grasping an object requires the hand to adapt to the shape of the object, and the development of strength will be directly related to the volume of the object. Thus, all the structures of the hand participate physiologically.

Considering the progressive nature of DMD, health professionals and researchers should use instruments that are capable of monitoring disease progression. In the evaluation of patients with reduced handgrip strength, pneumatic instruments, such as the modified sphygmomanometer, seem to be more advantageous than others, because they allow for the control of the resistance imposed [28], and they are more lightweight [18], easier to manipulate during the test [32], and have a manometer that is graduated in small intervals [19]. Thus, the modified sphygmomanometer seems to be more efficient for the evaluation of handgrip strength of patients with DMD than the dynamometer.

The main limitation of this study is the limited sample size, mainly across the longitudinal evaluations. Sample loss across evaluations is a common occurrence in follow-up clinical studies [33]. It is important to highlight that the patients who completed all evaluations of the study were not weak and did not present a lower score for the MFM dimension 3. Although our Rehabilitation Center receives patients from various regions of the country, adherence is not consistent. Another limitation is the absence of intra- and inter-rater reliability and agreement study. Although three measures for the dynamometer and the modified sphygmomanometer were obtained for each participant at four different evaluations, the mixed-effects model allowed the estimation of inter- and intra-individual variability to calculate the statistical inferences. However, obtaining a measure of variability of the differences which would allow the calculation of a measure of effect size, such as Cohen’s d, becomes a difficult task [34]. For this reason, we used the absolute differences between the means of the evaluations as an estimate of effect size, considering the known limitations of *p*-values [35]. Caution is still needed when this device is used by different raters. In conclusion, this study demonstrates that the modified sphygmomanometer seems to be more appropriate than the dynamometer for measuring handgrip strength in all stages of DMD.

## Conclusion

The results of this study indicate the potential for the modified sphygmomanometer to become a more suitable instrument for measuring handgrip strength of patients with reduced handgrip strength, such as DMD. Future study is needed to verify the reliability of the modified sphygmomanometer in patients with DMD.

## Data Availability

The data sets used and analyzed during the current study are available from the corresponding author, upon reasonable request.
